# Research progress in cell therapy for oral diseases: focus on cell sources and strategies to optimize cell function

**DOI:** 10.3389/fbioe.2024.1340728

**Published:** 2024-03-07

**Authors:** Jing Wang, Zeqing Zhao, Kai Yang, Yuxing Bai

**Affiliations:** Department of Orthodontics, School of Stomatology, Beijing Stomatological Hospital, Capital Medical University, Beijing, China

**Keywords:** cell therapy, oral diseases, cell function, optimization strategies, sources

## Abstract

In recent years, cell therapy has come to play an important therapeutic role in oral diseases. This paper reviews the active role of mesenchymal stem cells, immune cell sources, and other cells in oral disorders, and presents data supporting the role of cell therapy in oral disorders, including bone and tooth regeneration, oral mucosal disorders, oral soft tissue defects, salivary gland dysfunction, and orthodontic tooth movement. The paper will first review the progress of cell optimization strategies for oral diseases, including the use of hormones in combination with stem cells, gene-modified regulatory cells, epigenetic regulation of cells, drug regulation of cells, cell sheets/aggregates, cell-binding scaffold materials and hydrogels, nanotechnology, and 3D bioprinting of cells. In summary, we will focus on the therapeutic exploration of these different cell sources in oral diseases and the active application of the latest cell optimization strategies.

## 1 Introduction

The history of cell therapy can be traced back to the 19th century. Cell therapy has the potential to treat many currently intractable diseases through unique modes of action. The field continues to expand as research and investment continue (Zhang and Zhang, 2020). Cell therapy is a new type of therapy different from traditional drugs, with immune cells and mesenchymal stem cells (MSCs) being the two most promising types for disease treatment. Cellular therapies, whether investigational or marketed, offer therapeutic advantages to patients with a variety of medical conditions (Hoang et al., 2022). Cell therapy uses tissue culture methods to expand specialized cells to replace poorly differentiated cells in disease or to confer cells with stronger immune functions, to restore normal cellular function to the extent possible, and to achieve disease treatment (Helmy et al., 2020).

As a hot medical technology, cell therapy is characterized by precise targeting, high efficacy, and microscopic size (Rallis et al., 2021). Biological activities from these special “multipotent cells” of the body are leveraged, and after the necessary extraction, purification, and culture procedures, these cells are imported into the lesion site to change the local microenvironment (Romito and Cobellis, 2016). Through autocrine and paracrine pathways of cytokines, the self-repairing ability of the body is stimulated to the maximum extent, and *in situ* repair of damaged cells is completed, restoring damaged tissue and organ function to achieve the purpose of treating diseases at the cellular level (Han et al., 2022).

Experiments have demonstrated that, compared with the body’s normal repair process, the tissue repair rate after the application of therapeutic cells is tens or even hundreds of times faster, so that the damaged tissues of the body can be quickly restored in a short period (Martin and Nunan, 2015). The therapeutic process of cell regeneration therapy takes place at the molecular level and produces therapeutic effects on the diseased area at the cellular level. The treatment process requires only infusions and injections, which are less invasive than surgery. Not only is there less trauma and less pain, but there is also less stress and less risk involved (Harrell et al., 2019).

Cell therapy research has also focused on various oral diseases. Some researchers have shown that MSCs can induce periodontal tissue regeneration. By promoting osteogenic differentiation, MSCs can repair alveolar bone tissue defects and stabilize implants (Zhou and Shi, 2023). In an experimental canine trial, autologous bone marrow mesenchymal stem cells (BMSCs) and xenogenic periodontal ligament (PDL) MSCs showed beneficial effects on PDL reconstruction when used in combination with growth factors, fibrin glue, ephrin B2 (a membrane protein that regulates bone homeostasis), or porous biphasic calcium phosphate (Zhang et al., 2023). Regarding the strategy for pulp regeneration, studies have shown that autologous pulp stem cells, allogeneic stem cells, and autologous BMSCs have significant effects on pulp regeneration in canine models (Zhang et al., 2023). One study showed that the intra-articular injection of adipose tissue-derived mesenchymal stem cells (ADMSCs) combined with photobioregulation prevented degenerative knee osteoarthritis in rats (Ueyama et al., 2020). Therefore, it is expected that ADSCs and photobioregulation will be a therapeutic approach for the treatment of degenerative diseases of the temporomandibular joint.

In this review, we summarize therapeutic studies using different cell sources for different oral diseases and, for the first time, discuss the new advances of different cell optimization strategies for the treatment of oral diseases.

## 2 Sources of cells for cell therapy for oral diseases

### 2.1 Mesenchymal stem cells

Mesenchymal stem cells (MSCs), which can be successfully isolated from bone marrow, skin, teeth and other tissues, have direct osteogenic differentiation capability and low levels of immune rejection, and are widely used for regeneration in the oral and maxillofacial region.

#### 2.1.1 Bone marrow mesenchymal stem cells (BMMSCs)

BMMSCs are fusiform, non-hematopoietic stem cells isolated from bone marrow. Derived from the mesoderm, BMMSCs have a strong differentiation potential and can be induced to differentiate into muscle, cartilage, bone, fat, and nerve cells (Bhat et al., 2021).

BMMSCs can secrete a variety of immunosuppressive factors and have low immunogenicity. BMMSCs are the most suitable cell source for tissue regeneration due to their great differentiation potential. Utilizing BMSBCs for tissue engineering approaches, studies have shown that the vast majority of BMMSCs disappear within a relatively short time (on average 2–4 days) after injection into the host (Tan et al., 2022). However, they can also reprogram immune cells for a short period of time, which can influence disease development. BMSCs can be continuously transfused to treat disease to avoid the limitations associated with their retention in the host (Waldman et al., 2020).

#### 2.1.2 Adipose-derived mesenchymal stem cells (ADMSCs)

In 2001, Zuk isolated ADMSCs for the first time (Kuhbier et al., 2010). Subsequent studies showed that ADMSCs can differentiate into fat, bone, cartilage, nerve, skeletal muscle, myocardium, and islets under different induction conditions. ADMSCs can also secrete a variety of growth factors to promote angiogenesis. In recent years, due to the widespread existence of liposuction and the low operative complication of the host during the acquisition process, ADMSCs have shown some advantages over BMMSCs because of their easy acquisition, strong expansion ability, and sufficient supply, and have therefore become ideal seed cells (Yuan et al., 2022). ADMSCs also have the advantages of a high collection rate and few complications in the surgical donor area, making them an ideal source of seed cells for craniofacial repair and reconstruction. Craniomaxillofacial soft and hard tissue defects are common and frequent clinical diseases that seriously affect the appearance and function of patients. Restoration and reconstruction of such defects represent a comprehensive clinical problem involving many disciplines, for which there remain many problems to be solved. The development of tissue engineering has brought new ideas for craniomaxillofacial repair and reconstruction, and the source of seed cells represents the main problem in tissue engineering research. It has been reported that ADMSCs have a good application prospect in craniomaxillofacial fat tissue engineering, bone tissue and cartilage, nerve, skin, tooth and periodontal tissue regeneration, and facial repair (Zhang et al., 2020a).

#### 2.1.3 Dental pulp stem cells (DPSCs)

The concept of DPSCs, which are neural crest-derived ectodermal MSCs, was first proposed by Gronthos (Janebodin et al., 2011). These highly proliferative cells, isolated from human dental pulp tissue, are obtained upon enzymatic digestion of the extracellular matrix *in vitro* and are compared with bone marrow stromal stem cells (BMSCs). The authors showed that the two types of cells had similar immunophenotypes, but that the DPSCs had a higher rate of clone formation and were able to form scattered and high-density calcification bars after *in vitro* induction. DPSCs are adult stem cells with high proliferation, self-renewal ability and multiphase differentiation potential, which play an important role in dental pulp repair and tooth regeneration (Tsutsui, 2020). DPSCs express neural markers and secrete various neurotrophic factors, which can also play an important role in repairing and treating nervous system diseases through multiple pathways, especially parasecretory mechanisms (Fukuyama et al., 2020).

#### 2.1.4 Stem cells from human exfoliated deciduous teeth (SHED)

In 2003, for the first time, Miura reported the isolation of stem cells with multiple differentiation potential from human deciduous teeth, which were thus named stem cells from human exfoliated deciduous teeth (SHED) (Miura et al., 2003). SHED are similar to DPSCs in terms of cell morphology, cell surface markers, and multidirectional differentiation ability, with a spindle-shaped morphology and small volume (Qu et al., 2021). Human tooth pulp stem cells have better biological properties and differentiation potential than other odontogenic stem cells, especially in the direction of vasogenesis and neural differentiation. Additionally, their procurement is more convenient and their sources are more abundant (Okajcekova et al., 2020). SHED may represent a class of cells that has the ability to repair damaged tooth structures and induce bone formation, with the potential to treat nerve tissue damage and degenerative diseases. Previous studies have shown that compared to DPSCs, SHED not only have strong osteogenic differentiation ability, but also possess certain osteoclastic ability (Wang et al., 2018).

#### 2.1.5 Human periodontal ligament stem cells (hPDLSCs)

The hPDLSC subset was first discovered in 2004 (Maeda, 2020) when it was found to be derived from undifferentiated MSCs in the periodontal membrane. With excellent self-renewal and strong multi-differentiation potential, hPDLSCs have the advantages of easy availability, low tumorigenic risk, and no ethical controversy, and represent one of the most valuable cells for periodontal and bone tissue regeneration. When cultured *in vitro*, hPDLSCs can express numerous cementoblast/osteoblast markers and form mineralized nodules. hPDLSCs are the first choice of MSCs for periodontal regeneration, and play a key role in maintaining the stability of the periodontal ligament and repairing cementum tissue injury (Ouchi and Nakagawa, 2020). hPDLSCs are also key receptor cells for mechanical force during orthodontic tooth movement, and regulating the bone remodeling function of PDLSCs during orthodontic tooth movement represents a key scientific issue (Han et al., 2022a).

#### 2.1.6 Gingival mesenchymal stem cells (GMSCs)

GMSCs are MSCs of oral tissue origin that are isolated from gingival tissue. They not only are capable of cloning, self-renewal, multidirectional differentiation, and immune regulation, but also represent a promising source of cells for periodontal tissue engineering.

GMSCs have the advantages of homogeneity, non-tumorigenicity, easy separation, and phenotypic stability, and can be derived from oral treatments such as gingival plasty, implant therapy, and tooth extraction, without causing additional pain to patients. GMSCs have a higher proliferation rate than BMMSCs, and can be easily obtained in large numbers in a short period of time (Petrenko et al., 2020). GMSCs are homologous in primary culture, with no clear sign of aging after long-term culture. In contrast to BMMSCs, GMSCs can maintain their original shape (with an unchanged doubling time), do not lose cell markers, and have a normal karyotype (Petrenko et al., 2020).

As a newly discovered MSC, GMSCs have unique advantages over BMMSCs. Indeed, Tang L found that GMSCs had a greater ability than BMMSCs to delay the onset of rejection in a mouse skin transplantation model (Kamatani et al., 2022). GMSCs also have unique advantages compared to BMMSCs, including the ability to regulate the immune system, regenerate tissues, and play an important role in the treatment of some inflammatory diseases (Kamatani et al., 2022).

#### 2.1.7 Stem cells from the apical papilla (SCAP)

Young permanent teeth with periapical inflammation or apical abscess progress after conservative root canal treatment due to the role of SCAP, a new population of MSCs present in the apical papilla of young permanent teeth (Nagy et al., 2014).

As a tissue-derived MSC during development, SCAP exhibit potent self-replicating, self-renewing and tissue regenerative capabilities. As SCAP is derived from apical papillary tissue in the root of young permanent teeth during development and is the progenitor cell of pulp tissue, SCAP and DPSCs are histologically closely related and have certain similar properties. Therefore, we speculate that SCAP have more significant stem cell biological advantages than DPSCs, provide more abundant seed cells, and represent a better source of stem cells for dental tissue engineering (Shi et al., 2020). The *in vitro* biological characteristics of the two types of stem cells were compared in experiments, and it was confirmed that SCAPs have phenotypic characteristics and multidirectional differentiation potential, with a faster growth rate and greater cell activity and mineralization potential than those of DPSCs, thus representing a better source of stem cells (Chen et al., 2021b).

#### 2.1.8 Dental follicle stem cells (DFSCs)

Derived from neural crest cells, DFSCs are direct precursors of periodontal tissues and can differentiate into osteoblasts, periodontal membrane cells, and osteoblasts, playing an indispensable role in periodontal tissue development and possessing the potential for self-renewal and multi-differentiation. *In vitro*, DFSCs can be induced to differentiate into osteoblasts, adipocytes, neurons, salivary gland cells, duct cells, and cardiomyocytes under specific conditions (Jing et al., 2022). The dental follicle (DF) is a loose connective tissue derived from ectodermal mesenchyme that surrounds enamel organs and dental papillae during tooth development. Animal experiments have confirmed that DFSCs have the ability to generate osteogenesis *in vivo*, and that the combination of DFSCs and scaffold materials can repair large area bone defects *in vivo* (Wu et al., 2022). Moreover, DFSCs, which are easy to obtain and preserve, are promising seed cells for tissue regeneration.

### 2.2 Immune cell source

#### 2.2.1 T cells

Molecules and signaling pathways that are shared by the immune and skeletal systems play an important role in bone remodeling (Salhotra et al., 2020). The immune response depends on a complex collaboration between innate and adaptive immunity. T cells play a major role in adaptive immune response, initiating cellular immunity (Deets and Vance, 2021). T cells are thought to be persistent stimulators of bone destruction and may be involved in alveolar bone remodeling induced by orthodontic forces (Okamoto and Takayanagi, 2023). Tooth movement has been shown to be significantly reduced in immunocompromised mice with T cell deficiency, and blocking TNF-α, a key cytokine produced by type 1 T helper cells (Th1), inhibited the enhancement of orthodontic tooth movement and osteoclast generation in immunocompromised mice with T cell infusion (Farid et al., 2022). Thus, T cells are required for the orthodontic movement of teeth, and their role may depend on Th1-associated cytokines (Yan et al., 2015).

#### 2.2.2 Macrophages

Round or oval in shape, macrophages are the largest white blood cells and are phagocytes of the monocytic system. Monocytes are derived from precursor cells in the bone marrow, and macrophages are formed by the differentiation of monocytes in the blood, which remain in the bloodstream for only 12–48 h, before differentiating into different types of macrophages (Nasser et al., 2020). Macrophages not only have phagocytic and bactericidal functions, but can also eliminate ageing and damaged cells in the body and participate in immune function (Mosser et al., 2021). Studies have shown that macrophage-like cells found in the early stages of orthodontic tooth movement enhance bone and root resorption, and that macrophages polarize into various phenotypes, including classically activated M1 macrophages and alternatively activated M2 macrophages, which act as important immune cells that mediate inflammation (Wculek et al., 2022). Macrophages play a crucial role in diseases characterized by inflammation-mediated bone loss, such as rheumatoid arthritis and periodontal disease, and a previous study showed that systemic infusion of M1 macrophages increased the distance of orthodontic teeth (Mass et al., 2023).

#### 2.2.3 B cells

B cells are derived from hematopoietic stem cells and their differentiation process is regulated by a number of transcription factors (Ng, 2022). While B cells play a minor role in normal bone remodeling, activated B cells play an important role in inflammatory response diseases involving bone alterations (Bolamperti et al., 2022). In different disease settings, B cells can promote or inhibit osteoclasts, suggesting that B cells may limit bone loss under certain pathological conditions (Lee et al., 2021). Low levels of memory B cells in clinically healthy periodontal tissue play an important role in preventing bone loss from subclinical periodontal inflammation (Figueredo et al., 2019).

## 3 Role of cell therapy in oral disease

Numerous studies have shown that cells, through the secretion of cytokines, can activate various pathways to replace or repair damaged cells and exert a therapeutic role. Cells from different sources can produce different biological effects through different pathways, with broad prospects for use in dentistry. We review the function of cell therapy in major oral diseases from the perspective of clinical applications.

### 3.1 Role of cell therapy in bone and dental regeneration

MSCs have a variety of stable acquisition pathways and have become popular for regenerating bone and teeth. Their therapeutic efficacy is further enhanced by their ability to regulate other cellular functions and modulate systemic inflammatory conditions through cell-cell interactions or paracrine mechanisms (Evers et al., 2021). Currently, there are two main research directions for MSC-based bone and tooth regeneration: the recruitment of endogenous MSCs and the application of exogenous MSCs in cell therapy or tissue engineering (Rodriguez-Fuentes et al., 2021). As bone marrow-derived mesenchymal stem cells (BMMSCs) are closely involved in bone physiology and pathology, they are the most widely studied MSCs for bone regeneration. BMMSCs maintain bone homeostasis by differentiating into osteoblasts and regulating osteoclast activity and have shown good therapeutic potential in bone reduction and bone defects (Rodriguez-Fuentes et al., 2021). In patients with severe mandibular ridge resorption, researchers introduced BMMSCs with biphasic calcium phosphate scaffolds into resorbed alveolar ridges, inducing significant new bone formation sufficient for implant placement (Mohseni Salehi Monfared et al., 2022).

**FIGURE 1:**
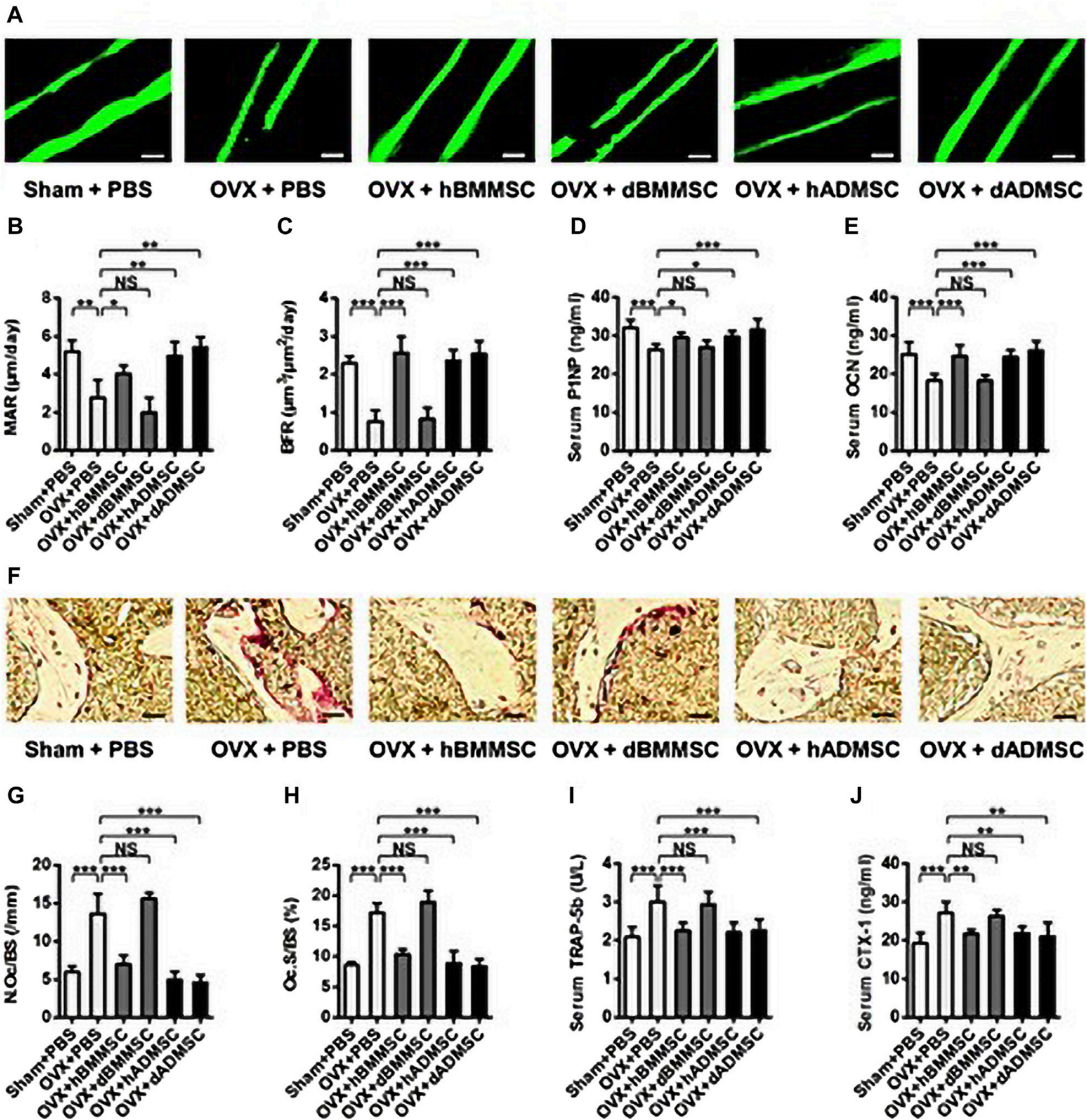
Bone remodeling changes after the infusion of Shamor osteoporotic donor-derived MSCs. **(A–C)** Representative images of calcein labelling **(A)** and parameters of bone formation rates **(B, C)** in distal femora harvested at D28 post operation. Bars: 50 μm; *n* = 3 per group. **(D, E)** ELISA analysis of serum levels of bone formation markers P1NP **(D)** and OCN **(E)**; *n* = 6 per group. **(F–H)** Representative images of tartaric-resistant acid phosphatase (TRAP) staining **(F)** and parameters of bone resorption rates **(G, H)** in distal femora harvested at D28 post operation. Bars: 25 μm; *n* = 3 per group. **(I, J)** ELISA analysis of serum levels of bone resorption markers TRAP-5b **(I)** and CTX-1 **(J)**; *n* = 6 per group. Data are shown as the mean ± SD. *p < 0.05, **p < 0.01 and ***p < 0.001. NS: Not significant (*p* > 0.05). Data were analyzed using ANOVA followed by Newman-Keuls *post hoc* tests. (Adapted from reference (Zheng et al., 2018), with permission).

Since their discovery by Zuk (2001), an increasing number of studies have demonstrated that ADMSCs have several advantages over BMMSCs, including stable *in vitro* growth dynamics, great potential for regenerative medicine, and less toxicity to the host. Recent studies have demonstrated the greater efficacy of ADMSCs compared with BMMSCs in repairing critical-size maxillofacial bone defects and constructing engineered bone grafts (Nauth et al., 2018; Zheng et al., 2018) (Figure 1). One study showed that functional differences between BMSCs and ADMSCs were due to differences in stem cells, energy metabolism, and antioxidant defenses. BMMSCs and ADMSCs were infused separately in osteoporotic mice. Serum markers of bone formation, such as procollagen-1 N-terminal peptide, osteocalcin (OCN), and type 1 collagen cross-linked C-terminal peptide, and staining with tartaric-resistant acid phosphatase (TRAP) were used to detect bone resorption activation. The results showed that ADMSCs were more effective than BMMSCs in inhibiting bone resorption. Therefore, the use of ADMSCs may be a more appropriate choice of treatment for the repair of bone defects in the maxillofacial region.

In one clinical trial, cranioplasty was performed on patients with skull defects using a combination of ADMSCs and beta-tricalcium phosphate granules (Mee et al., 2022). Among them, five patients who underwent cranioplasty were followed clinically and radiographically, and initial results were promising with no serious complications. However, at long-term follow-up, three patients underwent reoperation due to graft problems and two patients had satisfactory results. Further research is needed to investigate the use of MSCs in combination with beta-tricalcium phosphate granules for reconstructing cranial defects before proceeding to clinical trials.

There is an urgent need to fully restore the physiological function of teeth as dental problems caused by caries, periodontal disease, and pulp infection threaten oral and general health. Dental regeneration therapy is a new treatment strategy for dental tissue repair and whole tooth replacement. Dental stem cells and cellular activating factors have the potential to differentiate into dental tissues *in vivo* and *in vitro* and are therefore considered a pathway for dental tissue regeneration. Aggregates of SHED can regenerate intact pulp tissue in patients with pulp necrosis, if the pulp tissue is equipped with blood vessels and nerves (Li et al., 2022). Some studies have confirmed that after implantation of SHED, the root length of the recipient’s immature permanent teeth increased and the apical pore width decreased with increasing production of HIF1-a and VEGF, indicating that SHED-regenerated dental pulp maintained the continuous development of tooth roots as normal dental pulp and successfully regenerated dental pulp tissue.

Studies have also shown that DPSCs can be guided to differentiate into odontoblast cells through the Rho/RHO-related protein kinase signaling pathway, before functioning to repair defective teeth (Yan et al., 2010; Feng et al., 2016). Moreover, Zhang et al. (2020b) showed that neural crest cells derived from iPSCs can be differentiated into odontoblast-like cells under odontogenic induction with the administration of bone morphogenetic protein 4 (BMP-4), as identified by positive staining with odontogenic-related markers DMP-1 and dentin sialophosphoprotein (DSPP). DPSCs are expected to induce dental tissue regeneration. A recent study demonstrated that insulin-like growth factor 1 could be used to increase the size and cusp count of a bioengineered tooth by inducing enamel knot formation and proliferation and differentiation of dental epithelium and mesenchymal cells (Oyanagi et al., 2019).

The interaction between immune cells and stem cells is important in the process of tissue repair. Macrophages influence the differentiation of stem cells in the body and play a crucial role in tooth regeneration. Describing the interaction between immune cells and stem cells in the body is critical to understanding and improving the body’s repair mechanisms. Some researchers have used the *in vivo* injury model in combination with the macrophage and neutrophil consumption model to study the role of immune cells in restorative dentin formation (Bouchery and Harris, 2019). Previous studies have suggested that an increase in the endodontic Wnt pathway indirectly stimulates the polarization of M2 macrophages *in vivo*, thereby activating endodontic stem cells and encouraging restorative dentin formation (Neves et al., 2020).

### 3.2 Role of cell therapy in oral mucosal disorders

El-Menoufy injected autologous BMSCs suspended in phosphate-buffered saline under formocresol-induced oral ulcers. Compared with the control group, the expression of the genes for collagen and vascular endothelial growth factor was increased in the ulcers treated with MSCs. The experiment showed that the transplantation of MSCs helped speed the healing of oral ulcers (Kangari et al., 2020).

One of the most serious side effects of chemotherapy or radiotherapy is oral mucositis. Due to the immunoregulatory, anti-inflammatory, and regenerative properties of MSCs, they have a positive effect on treating oral mucositis. Zhang injected gingival mesenchymal stem cells (GMSCs) into mice with chemotherapy-induced oral mucosal inflammation and found that the GMSCs reduced the severity and incidence of ulcers and restored the nipple structure and intima and epithelial thickness (Wang et al., 2021b). The improved therapeutic efficacy of GMSCs may be attributed to their enhanced ability to engraft and survive at the site of injury, adaptability to hypoxia, and oxidative challenges (Kou et al., 2022). Therefore, GMSCs may prove to be a reliable treatment for oral mucositis following cancer treatment.

In addition, some researchers have demonstrated that Treg cells play an important role in maintaining the immune homeostasis of the oral mucosa. Oral inflammation in Rag1−/− mice was induced by the adoptive transfer of CD4^+^CD25^−^CD45Rb (high) T cells, and co-transfer of Treg cells with CD4^+^CD25^−^CD45Rb (high) T cells inhibited the development of oral inflammation in the mice (Wald et al., 2021). Treg cell therapy may be a promising new strategy for the treatment of oral inflammatory diseases, as it was shown to effectively inhibit oral mucosal inflammation in mice.

Oral submucosal fibrosis is a chronic, insidious disease associated with considerable morbidity (including pain and dysphagia) and an increased risk of malignant tumors (Qin et al., 2023). It is mainly caused by prolonged chewing of areca nuts and other damage to the oral mucosa. Stem cell therapy primarily promotes neovascularization through the release of cytokines and growth factors (paracrine effects), which help to stimulate the transformation of resident tissue stem cells into new fibroblasts. Sankaranarayanan injected 0.5–1 mL of bone marrow-derived stem cell concentrate into the lips, buccal mucosa, and tongue of patients under local anesthesia and found that when spicy food was consumed, mucosal softness was improved, the burning sensation was reduced, and the oral opening was significantly increased, demonstrating the efficacy of stem cell therapy for oral submucous fibrosis (OSMF) (Wang et al., 2021c).

### 3.3 Role of cell therapy in oral soft tissue defects

Oral soft tissues play an important role in the structure and function of the oral cavity, maintaining facial beauty and protecting the oral cavity from external factors. Repairing oral soft tissue defects caused by diseases or trauma is usually achieved by the transplantation of autologous mucosal tissue; however, such available autologous mucosal tissue is relatively rare, so other methods for repairing soft tissue defects must be sought (Matichescu et al., 2020). Relevant studies have shown that a cell-based approach to oral soft tissue regeneration is effective and safe (Tavelli et al., 2020).

In a randomized controlled hospital-based study, Mohammadi compared the effects of gingival adhesion width in patients receiving cultured gingival autografts with periosteal fenestrations or periosteal fenestrations alone (Mohammadi et al., 2022). Gingival fibroblasts were isolated from gingival biopsy specimens, expanded *in vitro*, and mixed with bovine collagen to provide a substrate and improve processing properties. Three months after surgery and graft placement culture, the results showed that the gingival grafts at the recipient site of keratinized gingiva increased significantly from the baseline compared with gingival grafts with periosteum fenestrations alone, and no postoperative adverse events were reported (Diar-Bakirly and El-Bialy, 2021). Therefore, autoculture of gingival grafts is safe.

### 3.4 Role of cell therapy in salivary gland dysfunction

Salivary gland dysfunction is usually a side effect of dry mouth after various medications, autoimmune diseases, or radiation therapy for head and neck cancer. Because saliva plays multiple roles in the mouth, reduced salivation can cause various symptoms, including mucositis, rampant dental caries, periodontal disease, and speech disorders (Jasmer et al., 2020). In particular, irreversible salivary gland dysfunction occurs in patients with radiation injury. Current treatments are palliative, and drug treatments, while speeding salivation of residual glandular acini in patients with mild or moderate injury, may not be effective in patients with severe injury (Zalewska et al., 2021).

Salivary gland dysfunction has become the focus of cell therapy research, usually using blood transfusion or the injection of isolated cells as drugs. Transplanted cells secrete molecules, such as growth factors and cytokines, to help the surrounding tissues regenerate (Marinkovic et al., 2023). It has been shown that the transplantation of salivary gland epithelial cells and MSCs from bone marrow promotes salivation in radiation-damaged glands (Ibrahim et al., 2017). Some studies have shown that soluble factors can also be detected in cell lysates transplanted into salivary glands, with an important role in supporting acinous cell regeneration and inhibiting apoptosis (Ibrahim et al., 2017), which may be similar to the mechanism of MSCs supporting the recovery of degenerated neurons.

### 3.5 Role of cell therapy in orthodontic tooth movement

In our team’s previous experiments to investigate the influence of stem cells on orthodontic tooth movement in mice, it was found that after injecting mBMMSCs into the tail vein of mice for 10 d, the tooth movement distance increased compared with that of the saline injection group. This mechanism may be due to the injected mBMMSCs being recruited to the compression side of the alveolar bone of the orthodontic tooth, and then promoting the number of osteoclasts on the compression side to increase significantly. Therefore, mBMMSCs positively promote orthodontic tooth movement in mice (Wang et al., 2021a).

Y. Yan’s research confirmed the importance of T cells in orthodontic tooth retention and movement. After orthodontic force was applied to wild-type (WT) mice, the number of TRAP-positive osteoclasts detected around the alveolar bone increased compared with that of immunocompromised mice. In addition, after the intravenous infusion of T cells, the number of TRAP-positive osteoclasts increased in the compression areas of the teeth of immunocompromised mice, suggesting that T cells are required for orthodontic tooth movement (OTM) (Yan et al., 2015).

S. Wilder’s work has shown that γδT cells are critical for orthodontic tooth movement. γδT cells are V-γ6+ cells located in the periodontal membrane that produce IL-17A and convert orthodontic mechanical forces into bone resorption in the OTM. Ablation of γδT cells strongly reduced the levels of neutrophils and RANKL in the PDL, which contributed to osteoclast production. In addition, mechanical forces were found to upregulate the expression of γδT cells and IL-17A, further confirming the role of γδT cells in OTM (Wald et al., 2021).

### 3.6 Role of cell therapy in temporomandibular joint (TMJ) disorders

A previous *in vitro* study demonstrated that adipose-derived stem cells cultured under chondroblast conditions in combination with polylactic acid (PLA) discs can be used in TMJ disc engineering to create a rep degenerative temporomandibular disc structure. Similar *in vivo* studies were conducted using ASCs cultured in PLA discs in rabbit TMJ. The use of ASCs in TMJ engineering promoted the formation of fibrocartilaginous TMJ disc-like tissue through high expression of TGF-β1 (Mieczkowska et al., 2018).

A study by Kim demonstrated the therapeutic potential of intra-articular injection of umbilical cord–derived mesenchymal stem cells (UC-MSCs) in the rabbit temporomandibular joint osteoarthritis (TMJOA) model. Transplanted UC-MSCs not only possessed anti-inflammatory effects similar to dexamethasone (DEX), but also improved cartilage and subchondral bone degeneration through upregulation of growth factors, extracellular matrix markers, and anti-inflammatory cytokines (Umer et al., 2022).

In addition, Zhang comprehensively demonstrated the pivotal role of matrix supplementation in the reversal of TMJOA. The results revealed no clear signs of cell proliferation after the transplantation of green fluorescent protein (GFP)-BMSCs, but the adequate stromal generation and clearance capacity of BMSCs could repair the cartilage of the osteoarticular processes. Furthermore, the repair effect was reversed when BMSCs were stopped from being delivered to arthritic joints (Antonarelli et al., 2023).

### 3.7 Role of cell therapy in periodontitis

*In vitro* studies have shown that aspirin induces periodontitis PDLSCs (P-PDLSCs) to express the generally controlled non-inhibitory protein 5 (GCN5), and then upregulates the expression of Dickkopf-associated protein 1 (DKK1), indirectly inhibits the Wnt-β catenin pathway, and ultimately promotes the osteogenic differentiation of P-PDLSCs. These findings suggest that aspirin enhances the function of PDLSCs and may be useful in regenerative periodontal health (Li et al., 2020c).

PDLSCs can also reduce the apoptosis and enhance the bactericidal activity of neutrophils through intercellular interaction and paracrine mechanism. This effect may be related to the synthesis of specialized pro-decomposable lipid mediators, such as decomposins, protectants, rosin and lipoxygenins, which are the main molecules responsible for coordinating the phase of inflammation resolution and the restoration of tissue homeostasis (Brennan et al., 2021).

In a study registered with the UMIN Clinical Trials Registry, autologous transplantation of PDLSCs in ten patients resulted in an improved depth of periodontal probing (PD), clinical attachment level, and radiographic bone height (alveolar bone regeneration). Moreover, when stimulated with appropriate growth factors, PDLSCs could express fibrogenic-like genes (e.g., Collagen-1 [COL1]), COL3, fibroblast-specific protein 1, periodontal-ligament-associated protein, and elastin, all of which improve the clinical application of periodontal regeneration (Queiroz et al., 2021).

### 3.8 Role of cell therapy in peri-implant inflammation

In a previous study, a model of peri-implant inflammation was established by implanting titanium implants in animals. GFP-ASCs were injected into the peri-implant capsule and exhibited a proliferative effect. The administration of ASCs reduced the thickness of the capsule, decreased the number of macrophages in the capsule, and reduced the mRNA level of fibrogenic genes in the peri-implant tissue. Angiogenesis was enhanced by trans-differentiation of ASCs into vascular endothelial cells and tissue hypoxia was alleviated (Park et al., 2023).

Another experiment investigated the effect of dexamethasone combined with DPSCs on peri-implant inflammation in diabetic Beagle dogs. Following treatment with dexamethasone spray combined with DPSCs, the bone height of the proximal and distal margins of the implants increased, the contents of TNF-α, IL-1β, and IL-6 in the gum crevicular fluid were significantly reduced, and the level of TGF-β3 significantly increased, which may be related to the reduction of the content of inflammatory factors and the upregulated level of TGF-β3 (Zhao et al., 2023).

## 4 Role of cell optimization in oral diseases

**FIGURE 2:**
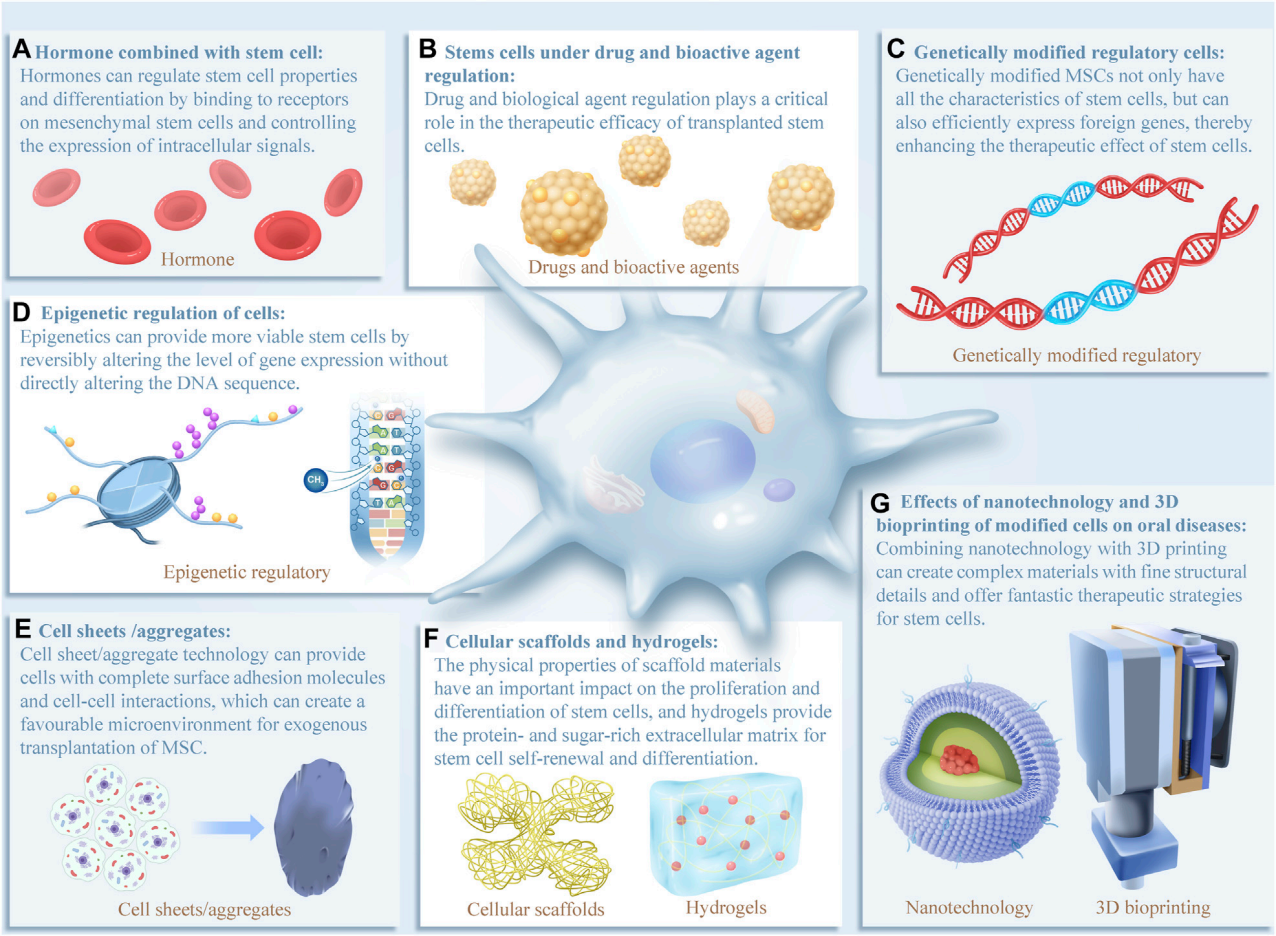
In recent years, various cell optimization methods have been extensively studied in oral clinical treatment, all of which have demonstrated good application prospects for oral disease diagnosis and treatment. **(A–G)** Schematic of cell optimization strategies and their effects on cells. The evidence that supports these effects will be discussed in more detail below. Reports have shown that the effects of different optimization strategies on cell function are extensive, highlighting the need to further explore the signaling pathways involved.

Recent studies have shown that different types of cell optimization methods can alter the cell microenvironment and activate different signaling pathways, which has new implications for clinical medical research. Different cell optimization methods may induce different biological effects in different ways, with potential applications in clinical medicine. In the subsequent sections, we aim to review the latest advances in various cell-optimizing strategies, highlighting their impact on cell function and relevant research for treating oral diseases (Figure 2).

### 4.1 Hormones combined with stem cell therapy

The binding of estrogen to the estrogen receptor (Erα and ERβ) on the cell membrane of PDL stem cells activates estrogen-responsive elements and modulates the expression of intracellular Notch signals (Gerhard et al., 1998). This promotes the proliferation and osteogenic differentiation of periodontal stem cells by increasing the expression of bone sialoprotein (BSP) and alkaline phosphatase (ALP), allowing periodontal fibers to repair and reattach to newly formed cementum and reconstructed alveolar bone (Borchers and Pieler, 2010).

By activating the androgen and estrogen receptors, a low (1 nM) dose of testosterone significantly stimulates the proliferation and migration of gingival fibroblasts *in vitro* by increasing the expression of JNK MAP kinase and/or WNT signaling, thereby promoting the healing of gingival tissue (Pathipati et al., 2011).

Researchers have shown that estrogen positively influences the osteogenic differentiation of odontogenic stem cells in periodontitis through regulating the expression of a variety of cytokines, including BMP-2, VEGF, and TGF-β. Additionally, other researchers have shown that phytoestrogens positively influence the osteogenic differentiation of odontogenic stem cells in periapical inflammation through activating the MAPK signaling pathway (Wang et al., 2022). Estrogens and phytoestrogens could be a new treatment approach for repairing bone defects in periodontitis and periapical inflammation (Chen et al., 2021a).

### 4.2 Influence of cells under drug and bioactive agent regulation on oral diseases

Drug regulation plays a critical role in the therapeutic efficacy of transplanted stem cells, creating favorable regenerative conditions that can optimize MSC-based bone and tooth regeneration therapy, which is a promising strategy (Zheng et al., 2019). Drugs and bioactive agents for regulating cells are shown in Table 1. Metformin increases the specific expression of the odontoblast proteins dentin sialophosphoprotein (DSPP) and dentin matrix protein 1 (DMP1) to promote the differentiation of dental pulp stem cells into odontoblasts, providing a scientific theoretical basis for using metformin to regenerate dental pulp (Weiss and Dahlke, 2019). IFN-γ inhibits fibroblast proliferation through reducing the expression of genes related to synthesis of Collagen I (Col1α1), Collagen Type I Alpha 2 Chain (Col1α2), and Collagen Type 3 Alpha 1 Chain (Col3α1), and reduces total collagen synthesis. Early use of IFN-γ prevents secondary deformities of the upper jaw caused by scar formation after cleft palate surgery (McKinnirey et al., 2021). In diabetics, by controlling the hyperglycemic microenvironment, hypoglycemic agents are used to promote MSC periodontal therapeutic effects through inducing MSC promoting M2 macrophage polarization and leading to reduced inflammation (Dong et al., 2018).

**TABLE 1:** Drugs and bioactive agents for regulating cells.

Type	Name	Effect	References
Hormone	Estrogen	Increases calcium deposition and expression of osteogenic genes	Gerhard et al. (1998), Borchers and Pieler (2010)
	Growth hormone	Stimulates bone formation and cartilage differentiation	Wang et al. (2022)
	Parathyroid hormone	Enhances bone morphogenetic proteins to stimulate calcium formation	Chen et al. (2021b)
Drugs	Metformin	Maintains the immunoregulatory capacity of mesenchymalstem cells (MSCs) under high glucose conditions	Weiss and Dahlke (2019)
	Aspirin	Inhibits the local pro-inflammatory state by downregulating IFN-γ and TNF-α	Tantry et al. (2021)
	Bortezomib	Inhibits the pathological inflammatory microenvironment	Ocansey et al. (2020)
Bioactive agents	IFN-γ	Initiates donor immunomodulatory properties and promotes bone formation	McKinnirey et al. (2021)
	Hypoglycemic agents	Promotes the periodontal therapeutic effect of MSCs by controlling the hyperglycemic microenvironment	Dong et al. (2018)
	Small molecule GSK-3β inhibitors	Promotes restorative dentin formation	Neves et al. (2020)
	Cytokine-inducible Src homology 2-containing protein (CISH)	Attenuates oral inflammatory pathways	Peng et al. (2020)

Different hormones, drugs and bioactive substances which regulate cells and their functions.

**FIGURE 3:**
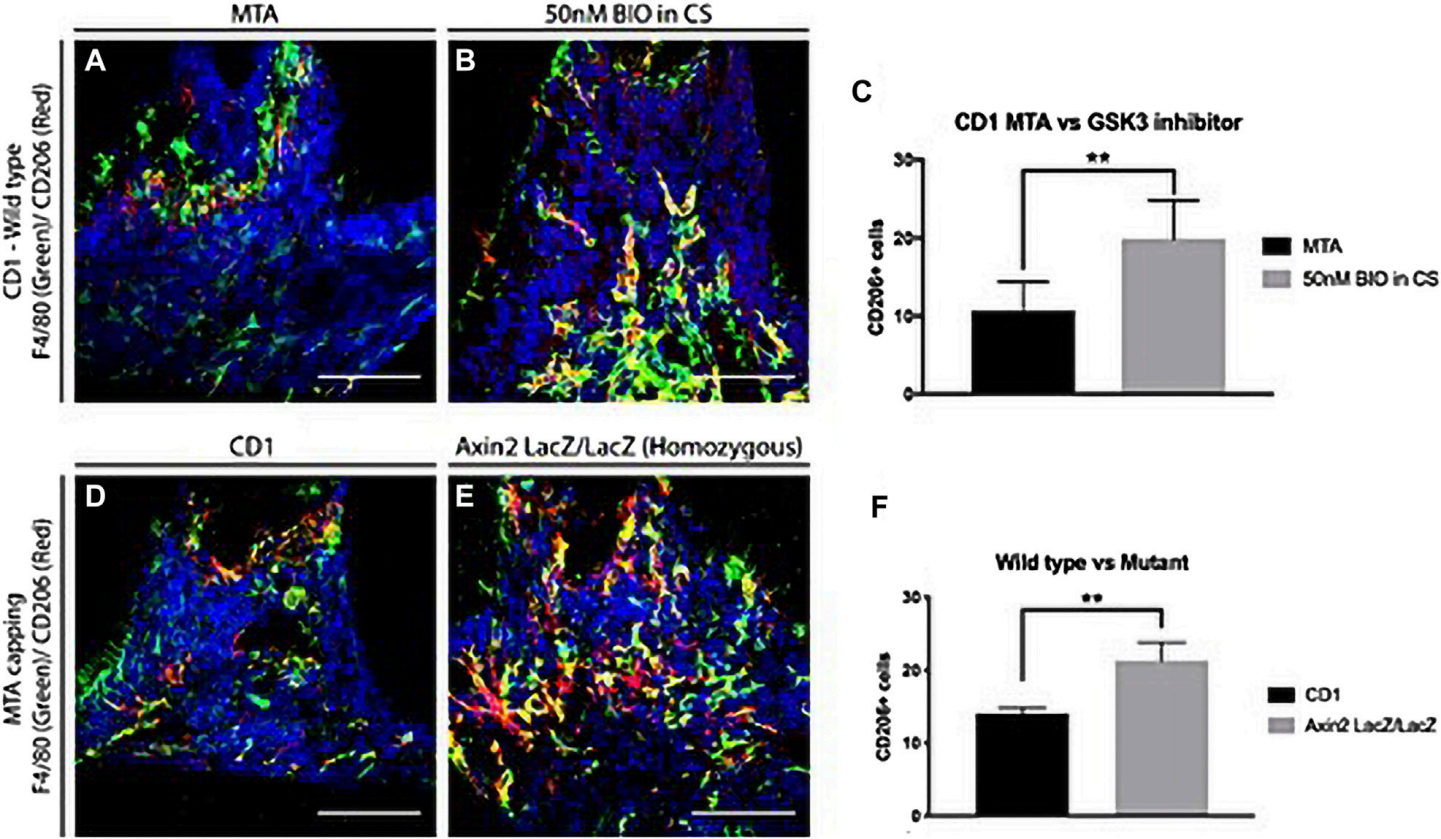
Anti-inflammatory macrophages in dentine repair. **(A–C)** Comparison of anti-inflammatory M2 macrophages 1 day after damage in CD1 wild-type mice upper first molars capped with either MTA or 50 nM BIO in clodrosomes or encapsomes. Staining for F4/80 (green—panel marker) and M2 marker CD206 (red) showed significantly fewer colocalized macrophages in mineral trioxide aggregate (MTA) capped molars than in those capped with 50 nM BIO in clodrosomes or encapsomes. **(D–F)** To confirm the results with the different capping materials, CD1 wild-type mice and Axin2LacZ/LacZ were damaged and capped with MTA. One day after damage, the number of M2 macrophages was analyzed. Staining for F4/80 and M2 marker CD206 confirmed an increase in M2 macrophages at the damage site following an increase in Wnt activity. **(C)** Unpaired t-test analysis: **p = 0.0033; (F) **p = 0.0014. Bars: 75 μm. (Adapted from reference (Neves et al., 2020), with permission).

Macrophages are required for pulp stem cell activation and proper restorative dentin formation. Pharmacological stimulation of the Wnt/β-catenin pathway using small molecule GSK-3β inhibitors enables macrophages to polarize into an anti-inflammatory state faster than stimulation with inert calcium silicate-based materials, increasing the number of anti-inflammatory M2 macrophages and thereby accelerating the stem cell effect in promoting restorative dentin formation (Neves et al., 2020) (Figure 3). These studies suggest that macrophage polarization from a pro-inflammatory to an anti-inflammatory phase can be accelerated by increasing Wnt levels in pulp cells during the early stages of pulp repair. Studies have shown that the GSK-3B inhibitor (a Wnt signaling pathway activator) also enhances the proliferation of human BMSCs via the β-catenin/PI3K/Akt signaling pathway (Peng et al., 2020). This could help develop drugs in the field of regenerative medicine, consistent with the abovementioned research.

### 4.3 Effects of genetically modified regulatory cells on oral diseases

MSC transplantation is a potential treatment strategy for mucositis. However, systematically infused MSCs rarely reach the site of inflammation, compromising their clinical effectiveness. Genetically modified MSCs not only have all the characteristics of stem cells, but they can also efficiently express foreign genes and better reach the diseased area, thereby enhancing the therapeutic effects of stem cells. Gene modification therapy involves the transfer of genetic material to introduce, inhibit, or manipulate specific genes to direct an individual’s cells to produce a therapeutic product or functional protein to treat a disease or disorder (Sayed et al., 2022). Therapeutic genes can be delivered directly to the patient by cell-based delivery with or without a scaffold matrix or indirectly by *in vitro* delivery. Gene therapy has significant advantages over traditional therapies (Paunovska et al., 2022). It can be used to replace mutated disease-causing genes, to alter the genetic structure of diseased cells, or to introduce genes that can boost the human body’s immune system (Bashor et al., 2022).

miR-26a overexpression rescued the impaired ability of estrogen-deficient murine MSCs in ectopic bone formation and skull defect repair by targeting both GSK3β and Smad1, which are negative and positive regulators of BMSC osteogenesis, respectively (Liu et al., 2018). Genetically engineered high expression of CXCR2 in MSCs enhanced the targeting ability of MSCs and accelerated ulcer healing by inhibiting the production of pro-inflammatory chemokines and radioactive reactive oxygen species (ROS) (Shen et al., 2018).

**FIGURE 4:**
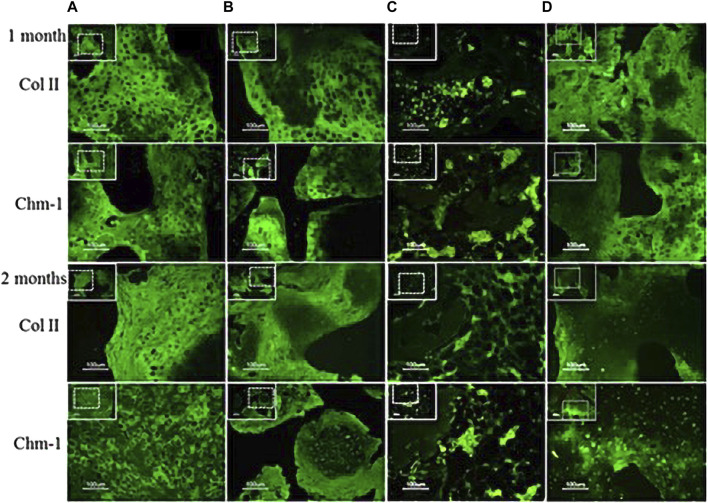
Analysis of chondrogenic protein expression (Chm-1) for neocartilage formation in the specimens after transplantion into nude mice. Specimens 1 and 2 months post transplantation: **(A)** chondrocyte–coral composites; **(B)** chondrocytes and MSCs coseeded into natural coral scaffolds in a ratio of 1:1; **(C)** MSC–coral composites; and **(D)** T-MSC–coral composites. Specimens were processed to analyze the distribution of Col II (green) and Chm-1 (green) in cells by immunofluorescence. Bars: 100 μm. (Adapted from reference (Chen et al., 2016), with permission).

Some researchers transferred chondromodulin-1 (Chm-1) adenovirus into BMSCs and applied the modified cells to engineered cartilage *in vivo*. The research results showed that Chm-1 was stably expressed in the engineered cartilage transplants. The newly formed tissue also showed strong immunohistochemical staining for Chm-1, and the chondroblast phenotype was well maintained by increasing the Wnt and BMP signaling pathways, indicating that MSCs modified with the Chm-1 gene could be used in cartilage tissue engineering, providing a laboratory basis for the repair of cartilage damage in the temporomandibular joint in the oral cavity (Chen et al., 2016) (Figure 4). Adipose stem cells transfected with lentivirus carrying chondromodulin are beneficial for repairing cartilage defects. This provides a theoretical basis for repairing temporomandibular joint damage, which is consistent with the abovementioned research.

Some researchers found that microRNA-935-modified BMSCs promoted the proliferation and differentiation of osteoblasts in osteoporotic rats through the secretion of extracellular vesicles carrying miR-935 targeting STAT1 (Morsczeck, 2022). In terms of long noncoding RNAs (lncRNAs), knocking out TUG1 inhibited the growth, proliferation, and invasion of OSCC cells by targeting the Wnt/β-catenin signaling pathway and inducing apoptosis of OSCC cells. Thus, knocking out TUG1 may be a new target for treating oral squamous epithelial cancer (Liang et al., 2017). MEG3 lncRNA is localized in the cytoplasm and interacts with enhancer of zeste homolog 2 (EZH2). Downregulation of MEG3 or EZH2 reduced H3K27me3 levels at the Wnt promoter, activating the Wnt/beta-catenin signaling pathway at the epigenetic level and subsequently promoting osteoblast differentiation of dental follicle stem cells (DFSCs) (Abuarqoub et al., 2023).

By studying the effects of gene modification on osteogenic differentiation and bone repair of MSCs, some scientists found that transplanting rat PDL cells modified with the human β-defensin 3 gene targeting of STAT1 into experimental periodontitis rats promoted bone repair in the rats (Li et al., 2020b). Therefore, genetically modified regulatory cells are expected to become a key focus in the field of oral disease treatment.

### 4.4 Influence of epigenetic regulation of cells on oral diseases

Epigenetics refers to reversible changes in gene expression levels without directly altering the DNA sequence (Banta and Richards, 2018). Epigenetic marks are heritable and relatively stable, and produce changes such as DNA methylation, histone modifications, promoter–enhancer interactions, and noncoding RNA–mediated regulation, which are often closely linked to the regulation of gene expression and involve many cellular processes (Cheung and Lau, 2005).

Genetic modifications or interference is the main strategy to control the fate of stem cells, through which cell source evaluation and cell modification can provide viable stem cells (Menche and Farin, 2021). Research has shown that the histone methylation inhibitor DZNep enhances the osteogenic potential of MSCs *in vitro* under pathogenic conditions by effectively derepressing Wnt signaling (Li et al., 2021). In addition, some researchers have shown that histone demethylases can promote the odontogenic differentiation of dental pulp MSCs through epigenetic regulation. They have also demonstrated that KDM6B can treat tooth structure regeneration and craniofacial defects through epigenetic regulation and modification by promoting mRNA expression of the odontogenic marker SP7 (Osterix, OSX) and extracellular matrix genes BGLAP (OCN) and SPP1 (OPN) (Xu et al., 2013).

Some scholars have confirmed that transplanting methyltransferase-like proteins (METTL 3) in *in vivo* overexpressed endothelial progenitor cells into mandibular callus can significantly enhance bone regeneration under distraction osteogenesis by regulating the PI3K/AKT pathway through m6A modification. Other studies have shown that METTL3 can enhance the stability of B lymphocyte mRNA mediated by m6A binding protein through m6A modification, and then inhibit the apoptosis and autophagy of chondrocytes in inflammation, so as to relieve TMJ (Wei et al., 2022). Other experiments have shown that knocking down AlkB homolog 3 (ALKBH3) can increase the level of m1A in transfer ribonucleic acid (tRNA) cells and reduce protein synthesis in cancer cells, thus inhibiting the progression of oral cancer (Liu et al., 2021). Besides, knocking out LSM1 homolog (LSM1)/nucleoside diphosphate-linked moiety X-type motif 5 (NUDT5) can inhibit head and neck squamous cell carcinoma (HNSCC) cell invasion and migration by inhibiting N7-methylguanosine (m7G) modification. These results help to broaden the understanding of M7G-mediated immune responses and identify new immunotherapeutic targets for HNSCC (Ying et al., 2023).

Post-translational modification of proteins is an important consideration in the study of oral diseases. RGS12 is an essential suppressor of oral cancer, which binds to phosphatase and tension homolog (PTEN) through the PDZ domain, controls the phosphorylation of PTEN, and further inhibits the AKT/mTOR pathway, thereby inhibiting the occurrence of oral squamous cell carcinoma. Therefore, the results suggest that RGS12 can be used as a potential therapeutic target and prognostic biomarker for oral squamous cell carcinoma (Fu et al., 2020).

Overexpression of acetyltransferase p300 in human dental pulp cells (HDPCs) has been shown to increase the levels of acetylated histone 3 lysine 9 (H3K9), which is located in the promoter and promoter regions of the odontogenic marker genes osteocalcin (OCN) and dentin sialophosphoprotein (DSPP) genes, both of which are significantly upregulated during the odontogenic differentiation process and promote odontogenic differentiation (Gronthos et al., 2014).

A previous study reported that the ubiquitin-specific protease 34 (USP34) plays a central role in the formation of tooth roots. Indeed, USP34-deficient dental pulp cells (DPCs) inhibited odontogenic differentiation with downregulation of nuclear factor I/C (NFIC), and overexpression of NFIC partially restores the impaired odontogenic potential of DPCs, as reported by Jiang et al. (2021). These results suggest that epigenetic modifications will identify new potential therapeutic targets for oral diseases.

### 4.5 Effects of cell sheets/aggregates on oral diseases

Cell sheet technology (CST) overcomes the shortcomings of traditional tissue engineering, demonstrating the innovation and benefits in the field. Compared with traditional tissue engineering techniques, CST cells have a high inoculation rate, abundant matrix, good biocompatibility, and ideal operability, which is conducive to cell transplantation and tissue reconstruction functions. CST has been successfully studied in periodontal tissue reconstruction, oral mucosal soft tissue defect repair, and maxillofacial bone defect repair (Roh et al., 2017). Cell sheet/aggregate technology provides cells with complete surface adhesion molecules and cell–cell interactions, which can create a favorable microenvironment for the exogenous transplantation of MSCs (Zheng et al., 2019).

**FIGURE 5:**
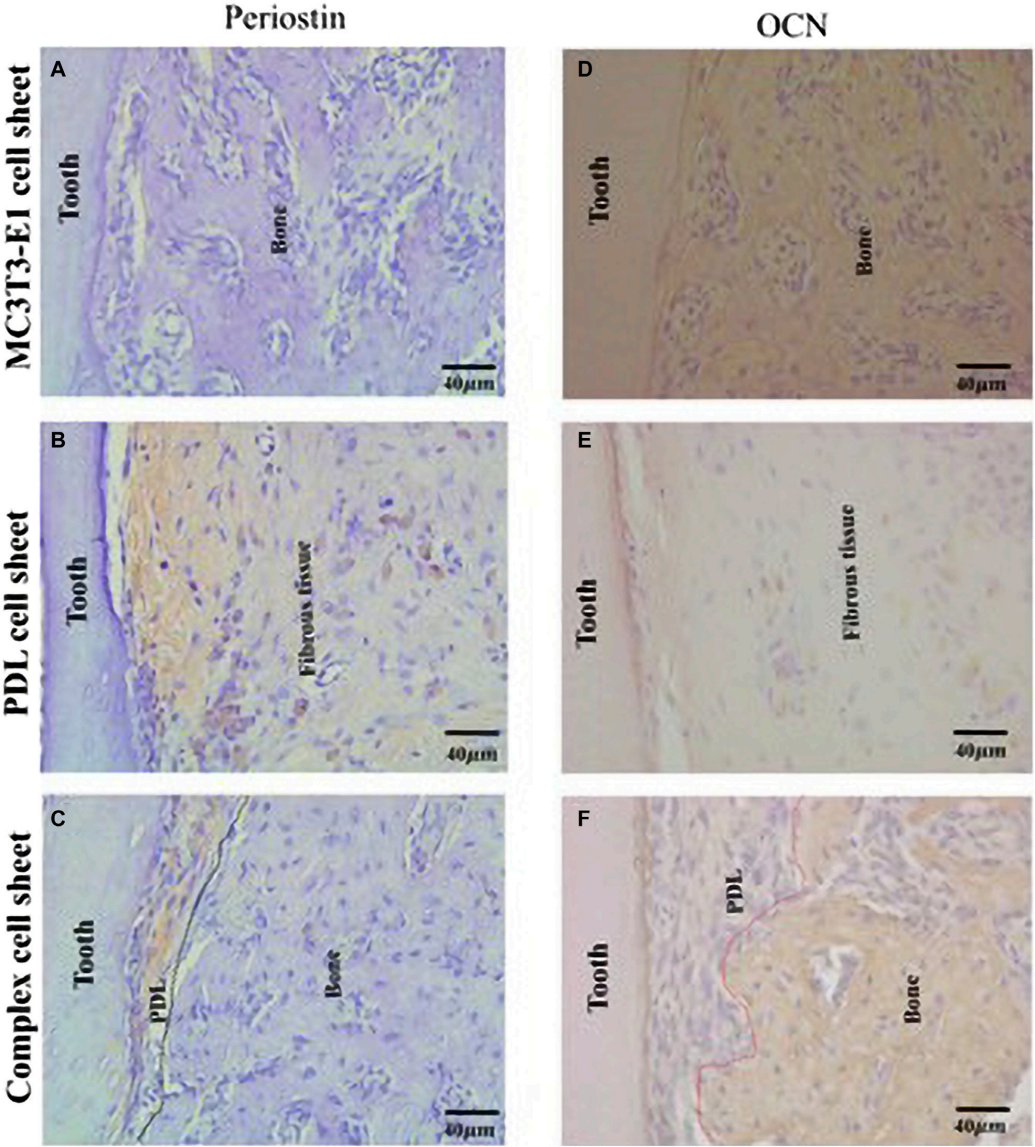
Formation of a periodontal tissue-like structure following ectopic transplantation of a complex cell sheet. Immunohistochemical staining of ectopic transplants. **(A)** MC3T3-E1 cell sheet transplants showed negative staining for anti-periostin antibody; **(B)** PDL cell sheet transplants shows positive staining for anti-periostin antibody; **(C)** complex cell sheet transplants showed positive staining for anti-periostin antibody in the PDL-like tissue space (PDL-like and bone-like tissue is demarcated using black dotted lines); **(D)** MC3T3-E1 cell sheet transplants showed positive staining for anti-mouse-OCN antibody; **(E)** PDL cell sheet transplants lacked positive staining for anti-mouse-OCN antibody; and **(F)** complex cell sheet transplants showed positive OCN staining at the bone-like tissue area (PDL-like and bone-like tissue is demarcated using red dotted lines) (Adapted from reference (Raju et al., 2020), with permission).

Three-dimensional periodontal tissue regeneration, using composite CST of bone and ligament, has been used for many types of tissue regeneration, including periodontal tissue, to transplant appropriate stem and progenitor cells to a single tissue regeneration target site (Raju et al., 2020). Composite cell sheets have anatomically regenerated bone ligament, functionally connecting PDL-like fibers to the tooth root and alveolar bone, and successfully achieved three-dimensional tissue regeneration for large areas of damaged tissue (Tang et al., 2014; Raju et al., 2020) (Figure 5). Osteoid tissue formation was observed in PDL cells and complex cells, and immunohistochemical expression of OCN and periosteum protein was detected in this region. Therefore, using bioengineered tissues to simulate anatomy can help regenerate damaged tissue on a large scale.

Akizuki developed PDL cell sheets using a temperature-responsive cell culture dish technique and a hyaluronic acid carrier, and significant new alveolar bone formation was observed after transplanting PDL cell sheets into animal models (Akiyama, 2021). Scientists transplanted oral mucosa cell sheets to a full-thickness skin excision wound in rats, thus revealing that the keratinocytes and fibroblasts grew faster in the oral mucosa of the experimental group than in the control wound (Roh et al., 2017). These results can be explained by the fact that oral mucosa cell tablets are rich in growth factors and cytokines, which can stimulate the growth and proliferation of keratinocytes and fibroblasts, thus accelerating the process of wound healing.

### 4.6 Effects of cellular scaffolds and hydrogels on oral diseases

**FIGURE 6:**
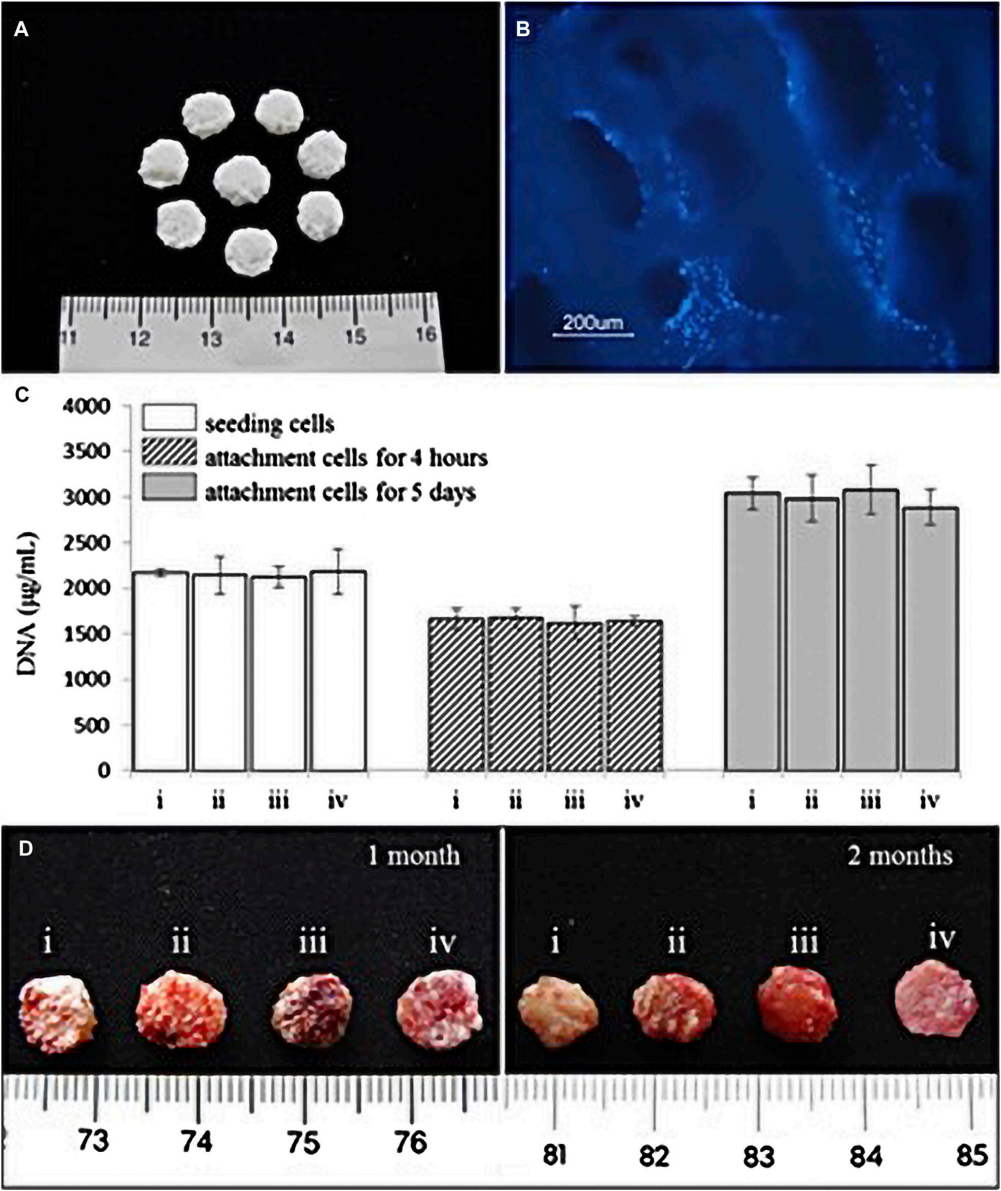
**(A)** Natural coral scaffolds (8 mm in diameter and 2 mm in height). **(B)** T-MSCs and coral scaffold complex. Fluorescence microscope examination showed the attachment of T-MSCs on the coral scaffold. Nuclei were visualized by Hoechst 33,342 staining. **(C)** Cell-seeding efficacy and proliferation on coral scaffolds (*n* = 4). Each scaffold was seeded with 2 × 10^6^ cells which were i. chondrocytes, ii. A 1:1 mixture of MSCs and chondrocytes, iii. MSCs, and iv. T-MSCs. The initial 2 × 10^6^ cells before seeding from each group acted as the control. There were no significant differences among the four groups (*p* > 0.05). **(D)** Representative macroscopic pictures of the cell–scaffold composites (i. chondrocyte–coral composites, ii. Chondrocytes and MSCs coseeded into natural coral scaffolds in ratio of 1:1, iii. MSC–coral composites, and iv. T-MSC–coral composites) removed from animals after 1 and 2 months. (Adapted from reference (Chen et al., 2016), with permission).

Scaffolds can be classified as acellular tissues, natural biomaterials (Figure 6), or synthetic biomaterials according to the source of scaffold materials (Garcia de Frutos et al., 2020). All cells are removed from animal or human tissues during the manufacturing process to create acellular tissue matrices, such as acellular adipose matrices (Chen et al., 2013). Furthermore, amniotic membrane has been proposed as a suitable biological scaffold for stem cell transplantation and proliferation, and has been shown to promote the antibacterial effect of stem cells in oral surgery and wound healing by inhibiting the production of LPS-upregulated IL-1α and Il-1β (Chang et al., 2010).

Natural biomaterials consist of proteins (collagen, gelatin, fibrin, and silk) and polysaccharides (agarose, alginate, hyaluronan, polylactic acid, and chitosan). Autologous adipose-derived multilineage progenitor cells and fibrin scaffolds isolated from subcutaneous adipose tissue were transplanted into periodontal defects using flap surgery. It was found that clinical adhesion improved compared with that of the control; the experimental group exhibited immunomodulatory effects through the upregulation of immunosuppressor genes and the downregulation of proinflammatory cytokines, and alveolar bone regeneration was induced (Venkataiah et al., 2019).

Polyethylene terephthalate (PET) and polyhydroxybutyrate-co-hydroxyvalerate (PHVB) can form porous, interwoven, rigid scaffolds, which allow for good cell infiltration and nutrient transport, enhance cell adhesion and growth, and promote oral hard tissue regeneration compared to their flat film counterparts (Sheikh et al., 2017).

Synthetic biomaterials include polymer-based biomaterials (e.g., polycaprolactone, polylactic acid, poly-lactic-co-glycolic acid, polyglycolide, poly-e-caprolactone, and poly-ethylene glycol) and ceramic-based biomaterials (e.g., hydroxyapatite, bioactive glass, and calcium phosphate). (Sampath and Reddi, 2020). An optimal β-tricalcium phosphate (β-TCP) dose of 5–10 wt% has been recommended to achieve appropriate mechanical stiffness and to support cell proliferation, neovascularization, and bone formation in collagen scaffolds. This dose range has been found to improve the mechanical properties of the scaffolds and promote the release of Ca^2+^ ions, improve cellular adhesion, accelerate cell differentiation and proliferation, and support vascularization. However, it is essential to note that as excessive Ca^2+^ ion release may suppress cellular activities, the optimal dose should be carefully tailored to achieve the desired balance between mechanical stiffness and biological performance (Miyazaki et al., 2021).

Bioactive glass has shown potential to promote bone regeneration and integration and serves as a promising material for oral tissue engineering applications. Bioactive glass has good biocompatibility and can promote the formation of a stable interface between bone cells and host tissues by releasing ions to form chemical bonds with surrounding tissues, promote the proliferation and differentiation of bone cells, provide a good growth environment for bone cells, accelerate bone regeneration, and improve the bone integration of self-tapping oral implants.

Hydrogels, which are networks of hydrophilic polymers, can be manufactured from natural biomaterials (e.g., collagen, fibrin, proteoglycans, and hyaluronic acid) or synthetic polymers (e.g., self-assembly peptide molecules or poly-ethylene glycol (Chen et al., 2016). Some studies have attempted to add dental stem cells to the hydrogel system to promote the regeneration of dental hard tissue. Through Schiff base reaction, Mohabatpour et al. prepared injectable hydrogels using oxidized alginate and carboxymethyl chitosan as cell carriers for tooth enamel regeneration. The hydrogels limited cell diffusion through a small pore size, thus promoting local cell proliferation and exhibiting enhanced mechanical strength through a high crosslinking degree. To provide better support for cells, experiments have proved that hydrogels can maintain the shape and vitality of dental epithelial cells within 14 days, and promote the regeneration of dental hard tissue by combining with dental stem cells, providing a new idea for the treatment of tooth injury (Roh et al., 2017).

### 4.7 Effects of nanotechnology and 3D bioprinting of modified cells on oral diseases

Three-dimensional printing technology and nanotechnology may help to enhance the properties of stem cells. Three-dimensional bioprinting is a combination of 3D printing technology, biology, and material science (Santoni et al., 2021). Biological materials, including cells and proteins, are printed directly into a confined space, thereby preserving the biological function and vitality of these materials (Santoni et al., 2021). Three-dimensional bioprinting can preserve the biological function, feasibility, and size of printed objects of biological materials. Three-dimensional bioprinting materials are composed of synthetic polymers, natural polymers, elastomers, ceramics, or hydrogels. A 3D-printed stent was made from a 3D model following a surgical plan predicted from CT and MRI data, which accurately filled defect spaces (Magin et al., 2016). Nanotechnology is the science and technology of building materials from single atoms and molecules and studying the properties and applications of these materials with structural sizes ranging from 0.1 to 100 nm (Laird et al., 2021). The combination of nanotechnology and 3D printing technology can create complex materials with fine structural details and offer fantastic therapeutic strategies.

Nanomaterials have unique optical, electronic, and mechanical properties and can therefore exert special biological effects (Wu et al., 2020). In a study of DPSCs, ROS clearance events in nanomaterials were associated with cell internalization-dependent length/diameter ratios. Moreover, when nanomaterials were smaller and longer in size, they were more easily phagocytosed and internalized by cells, thus allowing them to better penetrate cell membranes and bind to and remove intracellular ROS. This suggests nanomaterials can protect stem cells from environmental damage and enhancing their potential in tissue engineering and regenerative medicine (Liu et al., 2020). Nanocomposite membranes that can manipulate heterogeneous surface potentials enhance rapid bone regeneration by enhancing RGD (Arg-Gly-Asp) peptide binding and stem cell mechanical sensing, providing an alternative strategy for constructing novel dental stem cell scaffolds to repair intact mature bone structures (Li et al., 2020a). In addition, some scientists found that titanium-coated gold nanoparticles enhanced the osteogenic differentiation of human adipose-derived stem cells by increasing the Wnt/β-catenin signaling pathway (Kutrovskaya et al., 2019).

Three-dimensional printed biocompatible materials can simulate the microstructure and chemical composition of natural bone tissue, providing a suitable extracellular matrix environment for BMSCs to promote their growth and differentiation. Such materials can also release growth factors and cytokines to promote angiogenesis, provide sufficient nutrition and oxygen for BMSCs, and promote the healing of mandible wounds (Kim et al., 2021). Stem cells, cytokines, and cytokine-expressing carriers can be used as biological links for the direct printing of organ-like structures with bioprinters and can also be attached to magnetic nanoparticle materials to precisely fix the magnetic field at the target site (Deckers et al., 2023). New nanostructured materials can control cell fate and differentiation and can play a role in diagnosis, imaging, and targeted therapy of diseases, which is a new direction of stem cell therapy (Kong et al., 2021). Superparamagnetic iron oxide nanoparticles (SPIONs) can make cells magnetically controllable for site-specific enrichment. Furthermore, when SPIONs are attached to stem cells, they can be guided to specific target sites using external magnetic fields, allowing for precise delivery of therapeutic agents (Abdulghani and Mitchell, 2019).

## 5 Conclusion and perspective

We reviewed the treatment of various oral diseases with cells from different tissue origins and, for the first time, discussed new advances in various cell optimization strategies for the treatment of oral diseases (as shown in Table 2). Recent strategies for optimizing cells from different sources are the most promising and exciting in the world and play a promising and active role in advancing applications in dentistry.

**TABLE 2:** Optimisation strategies for cell function.

Method	Effect		References
Genetically modified regulation	miRNA	miR-26a promotes bone formation	Liu et al. (2018)
		micrornA-935 promotes the proliferation and differentiation of osteoblasts	Morsczeck (2022)
	High expression CXCR2	Accelerates ulcer healing by inhibiting the production of pro-inflammatory chemokines and radioactive reactive oxygen species	Shen et al. (2018)
	Chm-1 adenovirus	Maintains the chondroblast phenotype	Chen et al. (2016)
	LncRNA	TUG1 knockout induces OSCC cell apoptosis by targeting the Wnt/β-catenin signaling pathway	Liang et al. (2017)
	Human β-defensin 3 gene	Promotes periodontitis bone repair	Li et al. (2020c)
Epigenetic regulatory cells	Histone methylation inhibitor DZNep	Enhances osteogenic potential	Li et al. (2021)
	Histone demethylase KDM6B	Promotes odontogenic differentiation	Xu et al. (2013)
	m6A modification	Enhances bone regeneration under distraction osteogenesis	Wei et al. (2022)
	m6A modification	Relieves TMJ	Liu et al. (2021)
	m1A modification	Inhibits the progression of oral cancer	Ying et al. (2023)
	m7G modification	Inhibits HNSCC invasion and migration	Fu et al. (2020)
	Phosphorylation	Inhibits the occurrence of oral squamous cell carcinoma	Gronthos et al. (2014)
	Acetylation	Promotes odontogenic differentiation	Jiang et al. (2021)
	Ubiquitination	Promotes the formation of tooth roots	Tang et al. (2014)
Cell tablets/aggregates	Bone and ligament composite cell sheets	Regenerates the bone ligament and realizes the functional connection between the PDL-like fibers and the tooth root and alveolar bone	Akiyama (2021)
	PDL cell sheets	Promotes new alveolar bone formation	Roh et al. (2017)
	Oral mucosa cell tablets	Promotes keratinocyte and fibroblast growth	Venkataiah et al. (2019)
Cellular scaffolds and hydrogels	Acellular adipose matrix	Promotes antibacterial effects in oral surgery and wound healing	Sheikh et al. (2017)
	Adipose-derived multilineage progenitor cells on fibrin scaffolds	Induces alveolar bone regeneration	Su et al. (2015)
	Polyethylene terephthalate (PET), and polyhydroxybutyrate-co-hydroxyvalerate (PHVB)	Promotes oral hard tissue regeneration	Miyazaki et al. (2021)
	Cells on collagen scaffolds	Accelerates cell proliferation and supports vascularization	Chen et al. (2013)
	Cells on bioactive glass	Improves the bone integration of self-tapping oral implants	Garcia de Frutos et al. (2020)
	Stem cells on hydrogels	Promotes the regeneration of dental hard tissue	Roh et al. (2017)
	Oxidized alginate and carboxymethyl chitosan as cell carriers	Promotes tooth enamel regeneration	Liu et al. (2020)
Nanotechnology	Nanocomposite membranes	Enhances rapid bone regeneration	Kutrovskaya et al. (2019)
3D bioprinting modified cells	Titanium-coated gold nanoparticles	Enhances osteogenic differentiation	Kim et al. (2021)
	3D printed biocompatible and biodegradable nanomaterial scaffold	Promotes bone wound healing	Kong et al. (2021)
	Magnetic nanoparticle materials	Diagnosis, imaging, and targeted therapy of diseases	Abdulghani and Mitchell (2019)

Different cell optimization strategies and their roles, including gene modification regulatory cells, epigenetic regulatory cells, cell sheets/aggregates, cell binding scaffold materials and hydrogels, nanotechnology and 3D bioprinting modification of cells.

It should be noted that the translation of laboratory findings into clinical practice remains a challenge for any cell therapy for oral diseases. For example, genetically modified MSCs have the potential to provide new ideas and strategies for bone reconstruction and immune reconstitution after transplantation, which will be the future direction of cell therapy. By researching these issues, MSCs could have a better role in post-transplant bone and immune reconstruction. However, there are still many limitations. Notably, there are large differences in the *in vitro* amplification efficiency, and cultures take a long time, which cannot meet the needs of disease treatment promptly. These problems are limiting the use of genetically modified MSCs for transplantation.

Furthermore, our current understanding of the cellular and molecular mechanisms of combined hormone and stem cell therapy, drug regulation of cells, gene-modified regulatory cells, epigenetic regulation of cells, cell sheets/aggregates, cell-binding scaffold materials and hydrogels, nanotechnology, and 3D bioprinting of modified cells is still limited. Several outstanding issues require further investigation. First, the oral cavity is an extremely complex environment that is in a constant state of change. The effects of temperature, pH, oxygen levels, inflammation, microflora, and other factors on the cells of oral origin and their mechanisms are still unknown. The modified cells themselves are the second problem. Superphysiological numbers of cells are used to perform experiments. However, it is unclear whether physiological numbers of cells perform homeostatic or pathological functions in the body.

Technical bottlenecks in purifying, isolating, culturing, quantifying, and optimizing cells are a major limitation. For the promotion and development of the field of cell modification, there is a need for comprehensive research. The safety, homogenization, and standardization of modified cells should receive more attention in the future.
